# Supplementary material for: Deciphering the Impact of Defecation Frequency on Gut Microbiome Composition and Diversity
Source: Int J Mol Sci. 2024 Apr 25;25(9):4657. doi: 10.3390/ijms25094657 (PMC11083994; doi:10.3390/ijms25094657)
Supplement: Supplementary file 1 [file ijms-25-04657-s001.zip › ijms-2903934-supplementary.pdf]

Supplementary materials

Supplementary Figures S1-S7

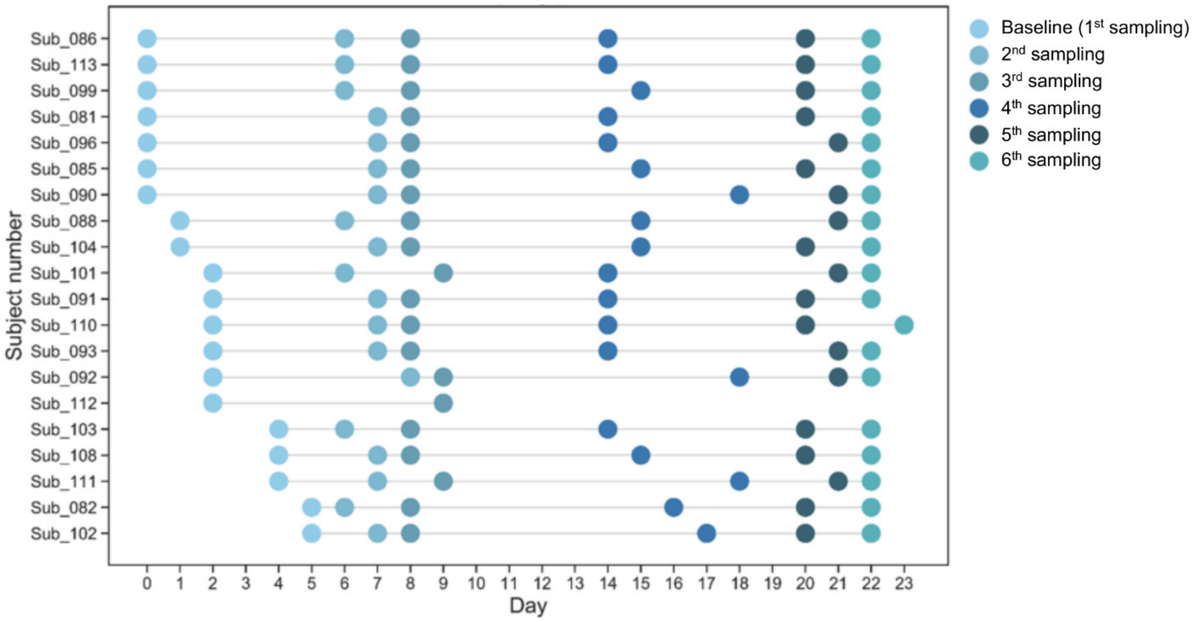

Figure S1. The schematic of sampling schedule across all participants. The division into defecation frequency group was based entirely on participants' self-reported defecation frequency per week, as obtained through a questionnaire and all participants were asked to collect fecal samples once or twice weekly based on their natural defecation frequency.

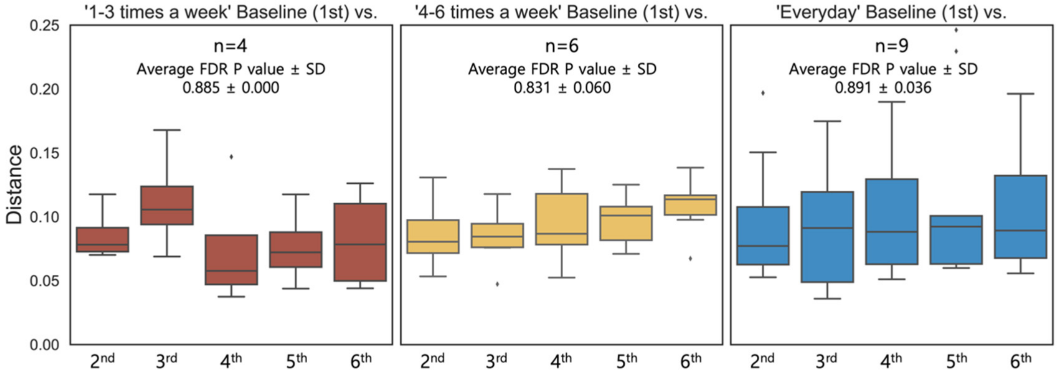

Figure S2. Intergroup distance across sampling timepoints within each defecation frequency group based on weighted UniFrac distance. Each sampling timepoint was compared to Baseline sample within each defecation frequency group. Mann-Whitney U test was performed for statistical significance (FDR, $p < 0.05$).

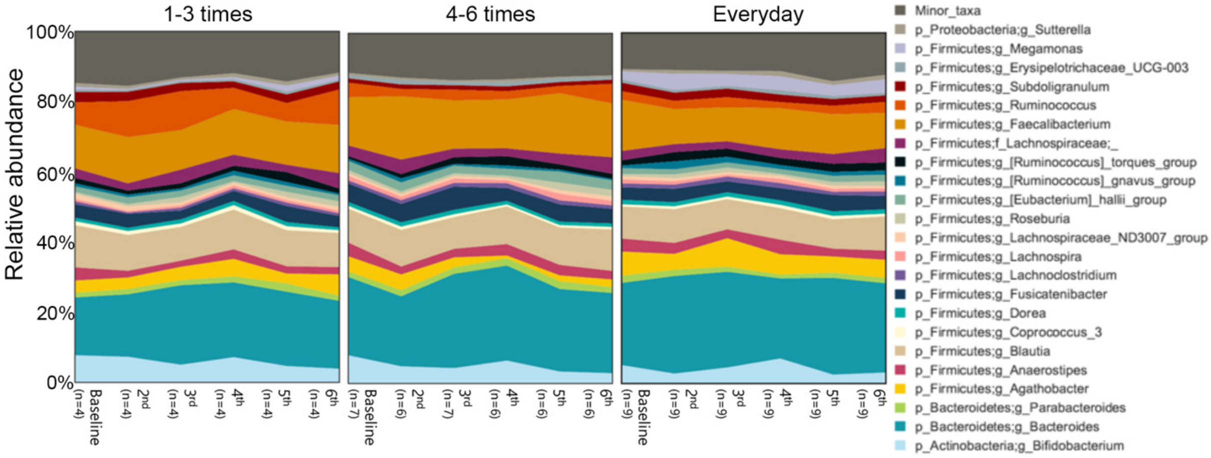

Figure S3. Changes in microbial composition at the genus level across different defecation frequency groups over six sampling points.

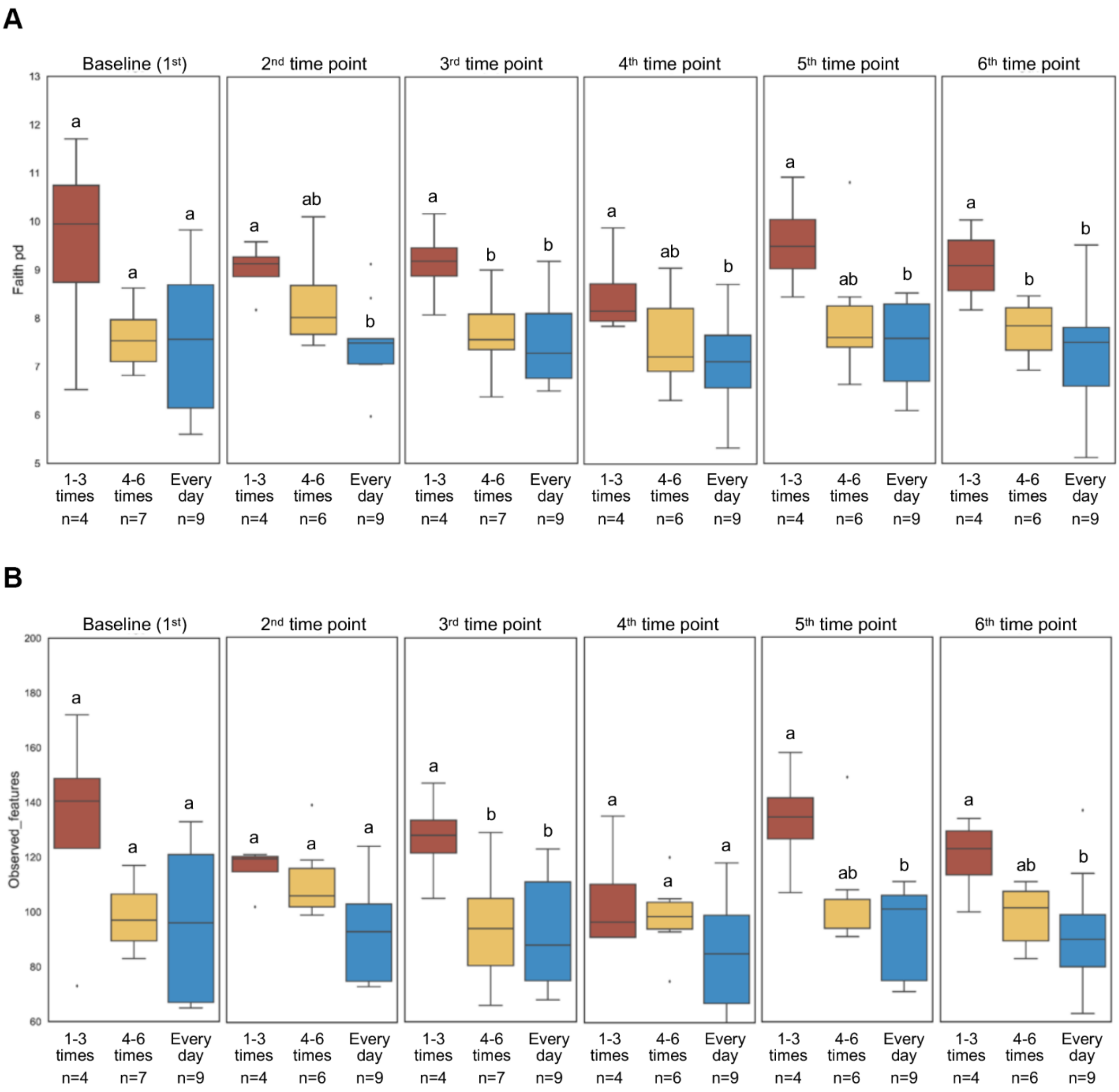

Figure S4. Alpha diversity of gut microbiota according to stool frequency at each sampling time point. Box plot of Faith's phylogenetic distance **(A)** and observed features metrices **(B)**. The inner line of the boxplots represents the median and the edges of the box are interquartile ranges (IQR). Statistical analysis was conducted using one-way ANOVA with Kruskal-Wallis test.

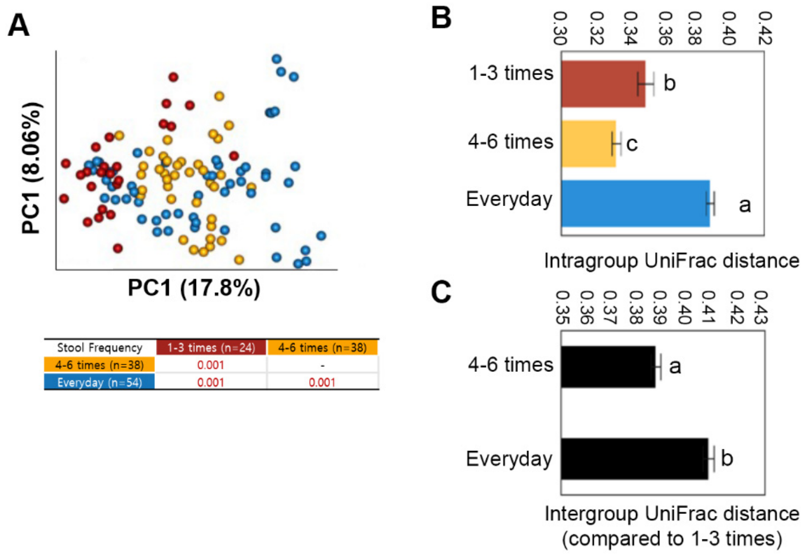

Stool Frequency	1-3 times (n=24)	4-6 times (n=38)
4-6 times (n=38)	0.001	-
Everyday (n=54)	0.001	0.001

Figure S5. Microbial diversity of gut microbiota using unweighted UniFrac distance according to stool frequency. **(A)** PCoA plot based on unweighted UniFrac distance. PERMANOVA was used to test dissimilarity. Intragroup **(B)** and intergroup **(C)** distances using unweighted UniFrac distance across three different groups. Data shown and error bars are mean ± SEM (B, one-way ANOVA with Tukey's multiple comparisons; C, non-parametric t-test, P<0.001). Different alphabets indicate significant differences between groups.

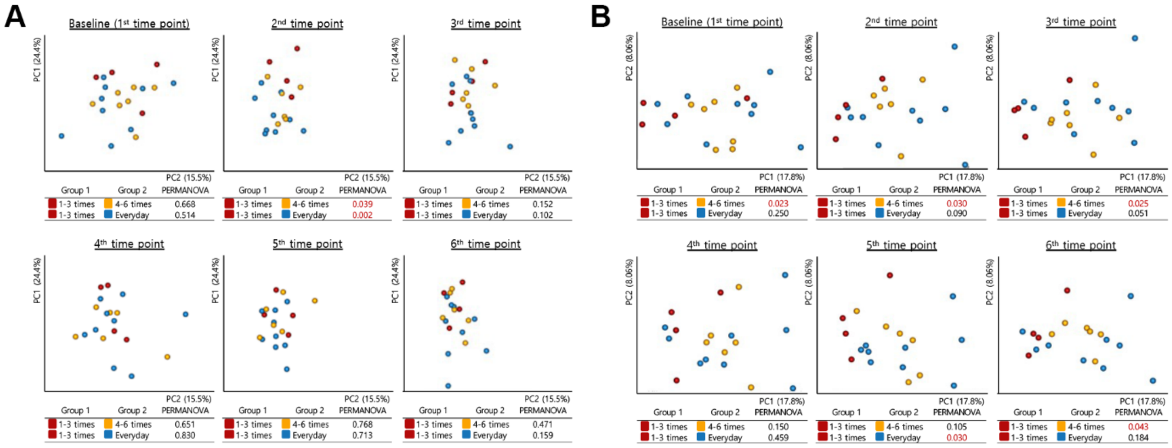

Figure S6. PCoA plot of bacterial communities distinguished by stool frequency at each sampling time point using **(A)** weighted and **(B)** unweighted UniFrac distances. PERMANOVA was used to test dissimilarity.

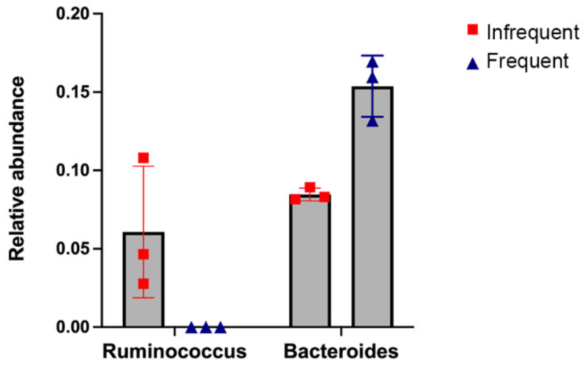

Figure S7. Relative abundance of two taxa, *Ruminococcus* and *Bacteroides*, among participants who were subjected to metabolite profiling (Infrequent, n=3 and Frequent, n=3).
